# Forensic Body Fluid RNA Typing: Evaluating the Roles of CE and dPCR Technologies

**DOI:** 10.3390/genes17070812

**Published:** 2026-07-16

**Authors:** Ana Mafalda Rocha, Maria Victoria Lareu, Catarina Xavier, Iva Gomes

**Affiliations:** 1 Instituto de Investigação e Inovação em Saúde da Universidade do Porto (i3S), 4200-135 Porto, Portugal; anar@i3s.up.pt (A.M.R.); cxavier@i3s.up.pt (C.X.); 2 Forensic Genetics Unit, Institute of Forensic Sciences (INCIFOR), Universidade de Santiago de Compostela, 15782 Santiago de Compostela, Spain; mvictoria.lareu@usc.es; 3 Institute of Molecular Pathology and Immunology of the University of Porto (IPATIMUP), 4200-135 Porto, Portugal

**Keywords:** body fluid identification, forensics, RNA profiling, mRNA, digital PCR, dPCR, RT-PCR, capillary electrophoresis, CE

## Abstract

The identification of body fluids in forensic science has increasingly relied on RNA-based approaches due to their tissue specificity and ability to provide probative information beyond DNA profiling. Among the analytical platforms used for RNA detection, capillary electrophoresis (CE) has traditionally served as the standard method in forensic laboratories, offering established workflows and effective multiplexing capabilities. However, recent studies in digital PCR (dPCR) have introduced a highly sensitive alternative that enables absolute quantification of target transcripts without reliance on standard curves, particularly beneficial for low-template and degraded forensic samples. This short review provides a focused comparative evaluation of CE- and dPCR-based workflows for RNA-based body fluid identification, emphasizing analytical performance and forensic applicability. Key parameters, including sensitivity, specificity, multiplexing capacity, tolerance to degraded RNA, and interpretation methods, are critically assessed. CE remains advantageous for multiplex assay design and routine forensic implementation due to its established infrastructure and throughput. In contrast, dPCR demonstrates superior sensitivity and quantitative precision, particularly in challenging forensic samples, but is currently limited by reduced multiplexing capacity and higher costs. The review highlights the methodological strengths and limitations of both platforms and examines their suitability for forensic casework. Overall, CE and dPCR are not positioned as competing replacements at the moment but rather as complementary technologies, with their combined or context-dependent use offering improved robustness in RNA-based forensic body fluid identification. Further validation and standardization studies are required to support broader implementation of dPCR in routine forensic workflows.

## 1. Introduction

The identification of biological material recovered from crime scenes is a valuable component of forensic investigations, providing contextual information that can assist in the reconstruction of criminal events and strengthen the evidential value of DNA profiles [1]. Traditional presumptive and confirmatory tests for body fluid identification, including chemical, enzymatic, and immunological assays, have long been used in forensic laboratories; however, many of these methods suffer from limited specificity, sensitivity, or high sample consumption [1]. In recent years, new molecular approaches, such as the use of RNA markers [2], have emerged as a potential and powerful alternative due to the tissue-specific expression patterns of RNA transcripts and their capacity to identify the cellular origin of biological stains with greater discriminatory power, particularly in mixtures [3].

RNA-based body fluid typing has become an increasingly important area of forensic genetics because it allows the simultaneous acquisition of contextual and genetic information from a single sample. Specific mRNA markers have been identified for a range of body fluids and tissues, including blood, semen, saliva, vaginal secretions, menstrual blood and rectal mucosa [4,5,6,7,8]. These markers can be incorporated into multiplex assays capable of analyzing several targets in parallel [4,9,10,11,12,13], thereby enhancing efficiency in forensic workflows. Despite early concerns regarding RNA instability, numerous studies have demonstrated that forensic RNA can remain sufficiently intact for analysis even in environmentally challenged or aged samples, supporting its practical application in casework [14,15]. The pioneering study by Juusola and Ballantyne in 2003 [16] demonstrated the potential of messenger RNA (mRNA) profiling as an alternative to conventional methods, establishing the conceptual basis for modern RNA-based forensic body fluid identification approaches. This study showed that specific mRNA markers are uniquely associated with different biological fluids, including blood, semen, saliva, vaginal secretions, and menstrual blood. Using a reverse transcription polymerase chain reaction (RT-PCR), the tissue-specific transcripts were successfully detected and accurately determined the origin of forensic stains [16]. Results further suggested that mRNA-based identification could improve forensic analyses by enabling multiplex testing, reducing sample consumption, and integrating efficiently with existing DNA profiling procedures. Unlike conventional serological assays, RNA profiling provides higher specificity and can be multiplexed with DNA analysis, allowing simultaneous acquisition of body fluid and donor genetic information from a single sample source. Early multiplex mRNA systems established the analytical foundation for forensic RNA typing and demonstrated that tissue-specific transcripts could remain detectable even in environmentally challenged or aged samples [4,16,17].

Compared with DNA methylation (DNAm)-based approaches, RNA profiling generally offers stronger tissue specificity and broader multiplexing possibilities. DNAm assays rely on tissue-dependent epigenetic modifications at CpG sites and have gained considerable attention because DNA is inherently more stable than RNA in forensic samples. Several collaborative exercises demonstrated the feasibility of DNAm-based body fluid typing and age prediction in forensic contexts [18,19]. DNAm markers are particularly attractive in highly degraded samples because methylated DNA regions often persist under conditions where RNA degradation is substantial. Furthermore, DNAm analysis can simultaneously provide additional intelligence information such as chronological age estimation, as demonstrated in studies targeting loci including *ELOVL2* [20]. However, DNAm assays often require bisulfite conversion, which can reduce template quantity and complicate workflows, while marker specificity for certain body fluids may be less distinct than transcriptomic signatures [21,22]. In contrast, mRNA markers frequently exhibit highly tissue-restricted expression patterns, improving discrimination between closely related fluids such as vaginal secretions and menstrual blood [23].

MicroRNA (miRNA)-based approaches represent another important molecular alternative. miRNAs possess intrinsic advantages for forensic applications because their short size and association with protein complexes confer enhanced stability relative to mRNA under environmentally challenging conditions. Consequently, numerous studies have investigated tissue-specific miRNA signatures as biomarkers for body fluid identification, demonstrating promising discriminatory performance across a range of forensic tissues and sample conditions [2,24], as well as promising discriminatory potential across forensic tissues [25,26]. Nevertheless, miRNA assays may exhibit lower tissue specificity than mRNA profiling because many miRNAs are expressed across multiple tissue types or body fluids with varying abundance levels rather than absolute specificity. Consequently, reliable forensic interpretation often requires larger marker panels and multivariate classification approaches [27,28]. Consequently, combined mRNA/miRNA strategies have emerged to capitalize on the specificity of mRNA and the stability of miRNA simultaneously [9,26].

More recently, microbiome-based body fluid identification has emerged as a complementary molecular strategy. These approaches exploit the characteristic microbial communities associated with different body sites and fluids. Studies investigating saliva, vaginal secretions, and other biological traces demonstrated that bacterial signatures could contribute valuable contextual information for body fluid classification [29,30,31]. Microbiome profiling may offer advantages in circumstances where host nucleic acids are highly degraded or present in minimal amounts. However, microbial compositions are strongly influenced by environmental exposure, individual variation, hygiene practices, and temporal changes, potentially reducing reproducibility and interpretative reliability in forensic casework [2,10,29,32,33]. Therefore, standardization of microbiome workflows remains an ongoing challenge [31,32].

MPS technologies have substantially expanded the analytical capabilities of RNA-based forensic typing. Targeted RNA sequencing permits simultaneous interrogation of large biomarker panels, improves sensitivity for low-template samples, and enables donor association through coding-region SNP analysis [34,35,36,37]. In comparison with traditional CE-based fragment analysis, sequencing approaches provide richer transcriptomic information and greater multiplexing capacity, although they require more sophisticated instrumentation, bioinformatic analysis, and validation procedures. Importantly, RNA sequencing also facilitates discovery-driven approaches, enabling the identification of novel biomarkers through whole transcriptome or miRNome analyses [38,39]. Despite the emergence of alternative molecular approaches, RNA profiling remains one of the most mature and operationally feasible tools for forensic body fluid identification. Extensive collaborative exercises conducted, for example, by EDNAP [40,41,42,43], EUROFORGEN [36,44,45], and FoRNAP [46], have demonstrated reproducibility, robustness, and applicability across multiple laboratories and sample types [36,40,41,42,43,44,45,46,47]. Nevertheless, no single molecular strategy currently satisfies all forensic requirements regarding sensitivity, specificity, degradation tolerance, multiplexing, cost-efficiency, and ease of interpretation. Consequently, current trends increasingly favor integrated or complementary approaches that combine RNA profiling with DNAm, microbiome analysis, or advanced sequencing technologies to maximize evidential value and improve interpretative confidence in forensic body fluid identification.

The analytical performance of RNA body fluid identification is strongly influenced by the detection platform employed. Capillary electrophoresis (CE) has traditionally been the standard analytical approach in forensic RNA typing [4,5,41,42,43,45,48]. CE-based workflows, commonly coupled with a reverse transcription polymerase chain reaction (RT-PCR), allow multiplex amplification and fragment separation with relatively high throughput and compatibility with existing forensic laboratory infrastructure [4,11,40,41,42,49,50,51,52]. The widespread implementation of CE in forensic DNA analysis has also facilitated the adaptation of RNA typing assays into routine workflows [53]. Nevertheless, CE-based methods rely largely on relative signal interpretation [48] and may experience limitations when analyzing low-template, highly degraded, or mixed forensic samples [45].

More recently, digital PCR (dPCR) has emerged as a promising alternative for forensic RNA analysis [10,54,55,56]. Unlike conventional PCR approaches, dPCR partitions a sample into thousands of independent reactions, enabling absolute quantification of nucleic acid targets without the need for calibration curves [57]. This partitioning strategy substantially improves analytical sensitivity and precision, particularly in samples containing low RNA quantities or inhibitors commonly encountered in forensic evidence. In addition, dPCR demonstrates improved tolerance to degradation and stochastic effects, characteristics that are highly advantageous in forensic applications where biological material is often compromised. However, despite these benefits, current dPCR systems remain limited in multiplexing capacity relative to CE-based platforms and are associated with increased instrumentation and operational costs.

Given the growing interest in RNA-based forensic body fluid identification and the rapid development of molecular technologies, a critical evaluation of the available analytical platforms is necessary. This review exclusively examines the roles of CE and dPCR technologies in forensic RNA typing, focusing on their analytical performance, practical implementation, and suitability for forensic casework. Other RNA-based approaches, including RT-qPCR and RT-PCR with NGS readout, were not included because they follow different relative or sequencing-based quantification models that fall outside the scope of targeted endpoint and partition-based assays. By comparing the strengths and limitations of both approaches, this review aims to assess their current and future contributions to forensic body fluid identification and to highlight the potential for complementary integration of these technologies in forensic casework.

## 2. Materials and Methods

The literature review was conducted focusing mainly on publications related to RNA-based forensic body fluid identification workflows using CE and dPCR technologies. Study searches were performed using multiple electronic databases such as PubMed and ScienceDirect, together with conference proceedings and scientific resources available through the International Society for Forensic Genetics (ISFG). The final results of the literature review are compiled in the Supplementary Materials, in Tables S1–S3. The search strategy, keywords and resulting metric data used were as follows:

(i) Keywords: “forensic body fluid identification”, “BFID”, “RNA profiling”, “mRNA”, “capillary electrophoresis”, “digital PCR” and “droplet digital PCR”, with the year search ranging from January 2003 to May 2026. Despite the fact that all studies using dPCR identified for forensic body fluid identification so far have employed droplet digital PCR (ddPCR), the nomenclature digital PCR (dPCR) was maintained throughout the manuscript whenever applicable, as the main aim of this review was to compare the two methodological approaches—RT-PCR CE and dPCR—rather than to focus exclusively on the ddPCR platform.
(ii) Searches retrieved 146 and 230 records from PubMed and ScienceDirect, respectively (376 records in total). After duplicate removal (*n* = 153), 223 unique records remained for title and abstract screening.
(iii) Records were excluded (*n* = 192) if they did not focus on forensic body fluid identification, and did not involve (i) RNA profiling, capillary electrophoresis, PCR multiplexes, or digital PCR-based approaches; (ii) were review articles, guidelines, or methodological papers without original experimental data; or (iii) addressed unrelated forensic applications. Only articles published in English were included in this review.
(iv) The final list of studies focusing on the targeted technologies, RT-PCR CE (*n* = 25) and dPCR (*n* = 6), is provided in Table S1 (Supplementary Materials). The list of marker usage per paper studied in this review, appropriately filtered by method and assay development, is shown in Table S3 (Supplementary Materials).
(v) Publications identified with other approaches were collected to gauge BFID interest in the forensic community (Table S2, Supplementary Materials). These were considered to provide a comprehensive overview of the state of the art in forensic body fluid identification, regardless of the analytical technology employed. Furthermore, they were incorporated into the publication trend analysis (January 2003–May 2026). These data were used for chart development illustrating the cumulative growth of forensic RNA-based body fluid identification studies over time, enabling contextual comparison with the evolution of RT-PCR CE and dPCR methodologies.

## 3. Results

### 3.1. Research Trends and Biomarker Distribution in Forensic RNA Body Fluid Identification

**Figure 1:**
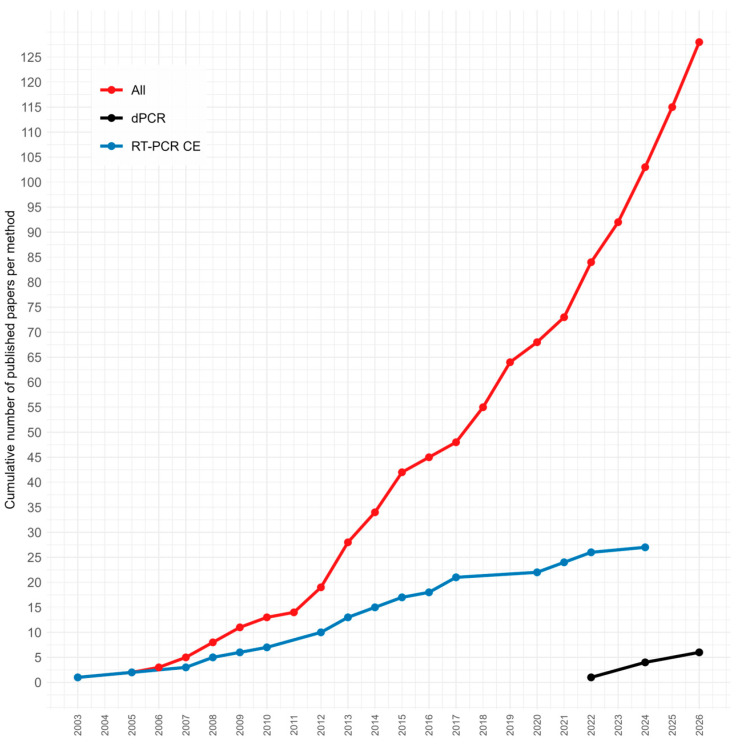
Cumulative number of published studies related to forensic RNA body fluid identification over time, showing overall publication growth (red), CE/RT-PCR-based studies (blue), and dPCR-based studies (black). CE-based approaches have dominated the field since the earliest studies and have shown sustained growth, whereas dPCR applications have emerged more recently and remain comparatively limited.

**Figure 2:**
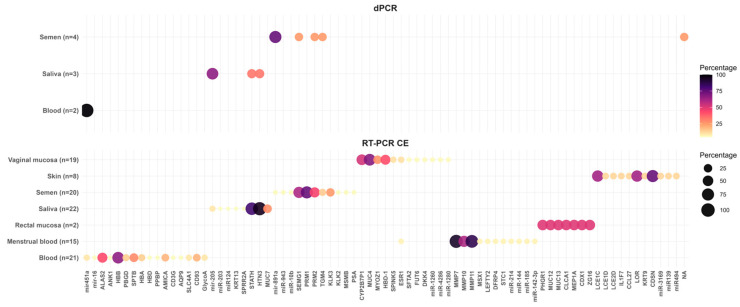
Representation of biomarkers identified through the literature review according to body fluid type, including blood, saliva, semen, vaginal mucosa, menstrual blood, and skin, for each methodology. Frequency of marker use in percentage of evaluated studies is given both in dot size and color intensity. The number of considered studies targeting each body fluid is displayed (*n*).

Analysis of publication trends and biomarker selection patterns can provide useful insight into the current state and developmental tendencies of forensic BFID research. Evaluating how frequently specific approaches and markers have been investigated helps identify dominant methodologies, commonly used targets, and emerging research directions within the field (Figure 1 and Figure 2). Figure 1 illustrates the chronological evolution of forensic RNA BFID studies. A progressive increase in the cumulative number of published studies over time reflects the growing scientific interest in RNA-based forensic applications (Figure 1). CE/RT-PCR-based approaches represented the predominant methodology throughout the analyzed period and showed sustained growth following the introduction of mRNA profiling for body fluid identification.

The overall number of publications increased gradually from the early 2000s onwards, coinciding with the introduction and subsequent development of RNA-based forensic methods (Figure 1). In contrast, dPCR-related studies emerged considerably later and currently account for a smaller proportion of the published literature, indicating that although dPCR has gained increasing attention, its implementation remains comparatively recent. Indeed, as other methodologies arise, such as microbiome markers, MPS sequencing of mRNA markers, and coding SNPs, among others, dPCR remains underexplored. Figure 2 represents body-fluid-specific biomarkers identified through the literature search conducted in this review and summarizes the frequency with which individual biomarkers have been investigated across published studies for different body fluids, including blood, saliva, semen, vaginal mucosa, menstrual blood, and skin.

Considerable variation in marker representation can be observed among body fluids and is represented in Figure 2 as the number of studies that have targeted a particular gene. Certain markers, such as established tissue-specific targets, have been repeatedly investigated and validated across multiple studies, whereas others appear less frequently and may represent more recently proposed or less extensively validated candidates (Figure 2). Interestingly, for saliva identification, *HTN3* (dPCR: 33%, CE: 95%) and *STATH* (dPCR: 33%, CE: 81%) have consistently been among the most frequently studied markers, having been explored and validated at an earlier stage in RT-PCR CE and more recently translated into dPCR methods, suggesting a strong association and sensitivity (Figure 2). Similarly, for menstrual blood identification, members of the matrix metalloproteinase (MMP) family, particularly *MMP10* (CE: 56%), *MMP11* (CE: 81%), and *MMP7* (CE: 88%), have represented the predominant mRNA targets used in forensic studies for this body fluid type (Figure 2), although not yet pursued in dPCR methods. Despite being moderately described in the literature (*n* = 8 studies), skin-associated markers are generally not included in most current BFID multiplex systems. This exclusion is primarily due to the limited additional discriminatory value, as skin tissue is commonly present across many biological samples, potentially resulting in redundancy within multiplex assay designs. Consequently, current BFID panels tend to prioritize markers with greater tissue specificity and higher evidential relevance for differentiating body fluids of forensic interest. The observed distribution likely reflects both historical and methodological factors influencing marker selection (Figure 2). Blood markers show a clear distinction of usage between methods, with dPCR techniques favoring the microRNA mir451a (dPCR: 100%), while the RT-PCR CE shows a broad use of different markers with a small tendency towards *HBB* (65%). Semen follows the same pattern, having mir-891a (dPCR: 75%, CE: 5%) as the most common marker in dPCR, followed by the well-known *SEMG1* (dPCR: 25%, CE: 60%), *PRM2* (dPCR: 25%, CE: 45%) and *TGM4* (dPCR 25%:, CE: 15%). In addition, the two markers, *PRM1* (75%) and *KLK3* (25%), are also found frequently in RT-PCR CE methods. Vaginal mucosa also shows three more relevant markers, *MUC4*, reaching the highest frequency (CE: 68%), followed by *CY2B7P1* (CE: 53%) and *HBD-1* (CE: 42%). A target that is increasing in interest in the forensic community, particularly relevant in (but not limited to) sexual assault cases of male victims, is the rectal mucosa (all markers CE: 50%). Here, the novelty of the application reflects the exploratory usage of the different markers. Biomarkers showing high representation in the literature may have been preferentially adopted because of reported specificity, reproducibility, compatibility with multiplex assays, or ease of incorporation into established forensic workflows. In contrast, less frequently investigated markers may indicate emerging targets requiring further validation. As a recent methodology applied to BFID, dPCR displayed not only less fluids targeted, but also a more modest number of tested markers, clearly favoring the microRNAs, which, being shorter (18–24 nucleotides), can be more stable and benefit dPCR design and detection. Nevertheless, a broad set of mRNA and microRNA markers for BFID is available in the literature, allowing forensic laboratories the flexibility to select and optimize marker panels according to specific requirements, sample characteristics, and routine workflows.

### 3.2. Capillary Electrophoresis vs. Digital PCR Workflows

**Figure 3:**
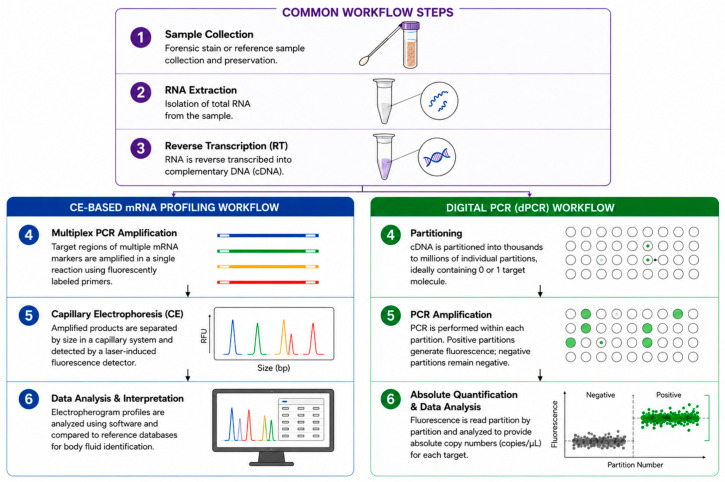
Schematic comparison of CE-based mRNA profiling and digital PCR (dPCR) workflows for forensic RNA body fluid identification. Both approaches share common initial steps, including sample collection, RNA extraction, and reverse transcription (RT) to generate cDNA. Following these stages, CE-based workflows involve multiplex PCR amplification, capillary electrophoresis, and electropherogram interpretation, whereas dPCR workflows include sample partitioning, PCR amplification within individual micro-reactions, and fluorescence-based absolute target quantification. CE provides high multiplexing capability and routine forensic applicability, while dPCR offers enhanced sensitivity and quantitative precision for challenging forensic samples.

Capillary electrophoresis (CE)-based RNA profiling remains one of the most established analytical approaches for forensic body fluid identification. In a typical CE workflow, body-fluid-specific transcripts undergo reverse transcription polymerase chain reaction (RT-PCR) amplification using fluorescently labeled primers, followed by separation of amplified products according to fragment size through CE (Figure 3).

Detection is subsequently achieved through electropherogram analysis (Figure 3), allowing simultaneous visualization of multiple transcript markers within a single assay. Progressive optimization of multiplex RT-PCR systems enabled the expansion of marker panels and increased throughput, facilitating implementation in forensic laboratories [4,22,32]. The widespread use of CE-based RNA profiling is largely attributable to its compatibility with existing forensic laboratory instrumentation and its ability to support multiplex analysis of numerous tissue-specific markers in a single reaction. Several multiplex systems have been developed for routine forensic applications, including expanded panels incorporating additional biomarkers and tissue-specific targets such as rectal mucosa markers [4,5,50,51,58]. The incorporation of alternative RNA classes, particularly miRNAs, has further broadened the range of detectable biomarkers and improved performance under environmentally challenging conditions [9]. Despite these advantages, CE-based methods remain inherently semi-quantitative because interpretation relies primarily on electropherogram peak profiles and threshold-based signal evaluation rather than direct molecular quantification [48,59]. Consequently, interpretation criteria may vary between analytical workflows and laboratories, particularly when evaluating weak signals, mixed samples, or compromised biological material [58,60]. Reduced sensitivity to trace quantities of RNA and difficulties associated with complex body fluid mixtures therefore remain important limitations in routine forensic implementation [54,61,62].

Digital PCR (dPCR) has emerged as a promising alternative methodology by enabling absolute quantification of nucleic acid targets through sample partitioning into thousands of individual micro-reactions [57,59,63]. Following amplification (Figure 1), each partition is classified according to fluorescence signal intensity as positive or negative, and target concentrations are subsequently calculated using Poisson-based statistical models [30,64]. Unlike CE-based fragment analysis, dPCR directly measures transcript copy number without reliance on calibration curves or electropherogram peak interpretation [57,59,60]. The principal advantages of dPCR are associated with its increased analytical sensitivity and enhanced quantitative precision [57,59,60]. Several studies demonstrated improved detection of low-copy-number targets, greater tolerance to PCR inhibitors, and superior performance in degraded or low-template forensic samples [57,61,65]. In addition, partition-based amplification reduces template competition and amplification bias, potentially improving identification of low-abundance transcripts and minor contributors in complex samples [57,59]. Recent digital PCR studies, in particular those using droplet digital PCR (ddPCR) platform technology, have demonstrated successful simultaneous identification of body fluid markers and improved evaluation of RNA degradation issues [54,56,66]. Nevertheless, important limitations currently restrict broader forensic implementation of dPCR workflows. Compared with CE-based systems, dPCR generally exhibits lower multiplexing capacity and requires more specialized instrumentation and increased operational costs [10,57]. Furthermore, forensic validation studies remain comparatively limited, and variability introduced during upstream processes, particularly reverse transcription, may still influence quantification accuracy [54]. Overall, CE-based and dPCR-based approaches represent distinct but complementary analytical strategies for forensic RNA BFID (Figure 3). CE-based profiling currently remains advantageous for routine high-throughput applications due to its established implementation and multiplex capabilities, whereas dPCR may provide particular benefits in challenging forensic scenarios involving trace, degraded, or inhibitor-rich biological material. Future comparative studies performed under standardized forensic conditions will be essential for defining the optimal applications and potential integration of both technologies within forensic RNA analysis workflows.

### 3.3. Forensic Applicability and Casework Relevance

The forensic applicability of any analytical method depends on its ability to generate reliable and interpretable results from casework-type samples. Biological traces recovered from crime scenes frequently contain low quantities of nucleic acids and may be affected by environmental degradation or inhibitory substances. In addition, forensic samples often consist of mixtures originating from multiple contributors and may contain highly imbalanced proportions of biological material, particularly in sexual assault cases. These conditions require analytical approaches that provide robust results and support reliable forensic interpretation, while also meeting admissibility standards [58]. The quantitative capabilities of dPCR may provide advantages in challenging forensic samples, particularly when low-abundance targets or highly unbalanced mixtures are present. Partition-based amplification can improve the detection of minor transcript populations and may facilitate the interpretation of complex biological mixtures. Such characteristics may be especially relevant in sexual assault casework, where epithelial and semen-derived RNA can occur in markedly different proportions.

Recent developments in data interpretation have also highlighted the potential of machine learning (ML) approaches for BFID analysis [29,38,39]. ML-based classification models have demonstrated high accuracy when applied to dPCR datasets, likely reflecting the reduced stochastic variation associated with absolute quantification [38,39,57,59]. Similar approaches have also improved the interpretation of CE RT-PCR data, particularly when quantitative information and mixture samples are incorporated into model training [63]. These developments suggest that advanced computational methods may further improve BFID interpretation regardless of the analytical platform employed.

From a casework perspective, both CE- and dPCR-based BFID approaches provide important contextual information by identifying the biological origin of forensic stains [64]. Such information can assist crime scene reconstruction, support or refute activity-level propositions, and guide subsequent DNA analysis strategies. Future developments are likely to focus on improved standardization, probabilistic interpretation frameworks, and integration with computational approaches, thereby increasing the evidential value and practical utility of RNA-based BFID in forensic investigations.

## 4. Future Perspectives and Conclusions

Future progress in forensic RNA-based body fluid identification is expected to be driven by continued methodological standardization, expanding molecular marker sets, and the integration of next-generation platforms. While CE-based RNA profiling remains the most established and widely validated approach in routine forensic laboratories, digital PCR (dPCR) is increasingly emerging as a complementary technology [10,48,54,58,65]. A key future requirement for the broader implementation of dPCR in forensic casework is systematic validation and interlaboratory standardization. Unlike CE-based systems, which benefit from extensive collaborative exercises and long-term routine use, dPCR-based forensic RNA applications remain comparatively under-validated. Consistent guidelines will therefore be necessary to address assay design, interpretation thresholds, reporting conventions, and quality assurance frameworks. In addition, pre-analytical factors such as RNA extraction efficiency and reverse transcription variability remain important sources of analytical variation and must be carefully evaluated within future validation studies [54,60]. Structured multi-laboratory collaborative studies will be essential to ensure reproducibility and forensic robustness. Future developments are also closely linked to the increasing number and refinement of RNA marker systems. Increasing availability of tissue-specific transcriptomic datasets supports the development of more discriminative and quantitative marker panels, including mRNA, miRNA, and other small RNA species. These markers are particularly relevant for dPCR-based workflows due to their suitability for absolute quantification and improved performance in degraded forensic samples [2,35,57,65,66]. In this context, marker selection is expected to shift from primarily qualitative classification toward quantitative decision frameworks based on transcript abundance thresholds and probabilistic interpretation models. In parallel, significant advances in MPS are expected to further develop BFID. Targeted RNA sequencing already enables simultaneous analysis of large biomarker panels and provides higher resolution of transcriptomic variation compared with CE-based fragment analysis [49,67,68]. In addition, MPS-based workflows support the identification of novel tissue-specific biomarkers and improve our understanding of transcriptomic complexity in forensic biological stains [35,67,68]. Also, the ability to integrate RNA sequencing with DNA-based analyses, including coding-region SNPs, allows for simultaneous body fluid identification and donor attribution from the same biological material [37,44].

Future developments in sequencing chemistry, library preparation, and bioinformatics are expected to further increase sensitivity, reduce costs, and improve compatibility with challenging forensic samples. Alongside RNA developments, DNA-based forensic technologies continue to evolve and increasingly complement RNA profiling strategies. Advances in DNA methylation analysis have expanded the range of biological information obtainable from forensic traces, enabling not only body fluid identification but also additional inferences such as age estimation [19,20,22,69]. Methylation-based approaches, particularly when combined with high-throughput sequencing or digital PCR platforms, offer promising alternatives for multidimensional forensic interpretation, especially in cases where RNA degradation limits transcriptomic analysis. However, challenges such as bisulfite conversion-induced DNA loss and marker-specific sensitivity constraints remain important considerations for routine implementation [2,21,22,29].

Overall, the future landscape of forensic body fluid identification is expected to move towards integrated multi-omics frameworks combining RNA profiling, DNA methylation analysis, microbial signatures, and sequencing-based technologies. Within this evolving framework, CE-based methods are likely to remain central for rapid multiplex screening, while dPCR- and MPS-based platforms provide complementary advantages in sensitivity, quantification, and information depth. The merging of these technologies, supported by improved standardization and bioinformatic interpretation tools, will be essential for maximizing evidential value (such as contextual information) and improving robustness in complex forensic case scenarios.

## Data Availability

Data generated for the present review are available in the Supplementary Materials.

## Supplementary Materials

The following supporting information can be downloaded at https://www.mdpi.com/article/10.3390/genes17070812/s1: Table S1: List of papers focused on this review for RT-PCR CE (*n* = 25) vs dPCR (*n* = 6) comparison. Table S2: List of publications focusing on other methods/markers/target (non-overlapping with Table S1) used for Figure 1 cummulative year chart on number of publications. Table S3: List of marker usage per paper studied in this review, appropriately filtered by method and assay development (Figure 2).

## Abbreviations

The following abbreviations are used in this manuscript:

BFID	Body Fluid Identification
CE	Capillary Electrophoresis
dPCR	Digital PCR
ddPCR	Droplet digital PCR
mRNA	Messenger RNA
miRNA	MicroRNA
DNAm	DNA Methylation
ML	Machine Learning
MPS	Massive Parallel Sequencing
RT-PCR	Reverse Transcription Polymerase Chain Reaction
